# Effect of Perfluorooctanoic Acid on the Epigenetic and Tight Junction Genes of the Mouse Intestine

**DOI:** 10.3390/toxics8030064

**Published:** 2020-08-28

**Authors:** Faizan Rashid, Saeed Ahmad, Joseph Maria Kumar Irudayaraj

**Affiliations:** 1 Biomedical Research Center in Mills Breast Cancer Institute, Carle Foundation Hospital, Urbana, IL 61801, USA; faizanr2@illinois.edu (F.R.); saeeda2@illinois.edu (S.A.); 2 Department of Comparative Biosciences, College of Veterinary Medicine, University of Illinois at Urbana-Champaign, Urbana, IL 61801, USA; 3 Department of Bioengineering, University of Illinois at Urbana-Champaign, Urbana, IL 61801, USA; 4 Micro and Nanotechnology Laboratory, University of Illinois at Urbana-Champaign, Urbana, IL 61801, USA; 5 Cancer Center at Illinois, University of Illinois at Urbana-Champaign, Urbana, IL 61801, USA

**Keywords:** PFOA, small intestine, colon, bioaccumulation, epigenetic toxicity, tight junctions

## Abstract

Perfluorooctanoic acid (PFOA) has been implicated in various toxicities including neurotoxicity, genotoxicity, nephrotoxicity, epigenetic toxicity, immunotoxicity, reproductive toxicity, and hepatotoxicity. However, information on the accumulation of PFOA in the intestine and its toxic effects on intestinal epigenetics and tight junction (TJ) genes is sparse. CD1 mice were dosed with PFOA (1, 5, 10, or 20 mg/kg/day) for 10 days, and its accumulation and induced alterations in the expression of epigenetic and tight junction genes in the small intestine and colon were evaluated using LC–MS and qPCR techniques. PFOA reduced the expression levels of DNA methyltransferases (*Dnmt1, Dnmt3a, Dnmt3b*) primarily in the small intestine whereas, in the colon, a decrease was observed only at high concentrations. Moreover, ten-eleven translocation genes (*Tet2* and *Tet3*) expression was dysregulated in the small intestine, whereas in the colon *Tets* remained unaffected. The tight junction genes Claudins (*Cldn*), Occludin (*Ocln*), and Tight Junction Protein (*Tjp*) were also heavily altered in the small intestine. TJs responded differently across the gut, in proportion to PFOA dosing. Our study reveals that PFOA triggers DNA methylation changes and alters the expression of genes essential for maintaining the physical barrier of intestine, with more profound effects in the small intestine compared to the colon.

## 1. Introduction

Perfluorooctanoic acid (PFOA) is an eight-carbon chain (C8) compound which belongs to a group of synthetic chemicals known as perfluoroalkyl and polyfluoroalkyl substances (PFASs). Since the 1950s, PFAS compounds have been widely used in the synthesis of various household and industrial products such as greaseproof papers, stain repellants, non-stick cookware, cleaners, carpets, aqueous film-forming foam and in wetting agents [1,2]. Among PFASs, PFOA has been used excessively because of its polymerization properties in the synthesis of various fluoropolymers used in the production of commercial products, such as Gore-Tex and Teflon [3].

PFOA has been detected in house dust, [4] surface and drinking water, [5,6], indoor and outdoor air [7], and animal tissues [8,9]. Humans are exposed to PFOA through diet, primarily eggs, fish products, poultry products, cereals, vegetables and drinking water, inhalation of dust particles, and in commercial products [5,10,11,12]. PFOA has strong carbon–fluorine bonds which makes it a highly persistent compound, and its bioaccumulation may eventually lead to adverse health effects [13]. Public concern of PFOA has increased since its detection in blood serum and breast milk of humans [14,15]. PFOA levels may remain high for an extended period of time owing to their long half-life of 2 to 3 years in human serum [16,17].

The bioaccumulation of PFOA in human serum and tissues has led to adverse effects on human health, such as an increase in levels of uric acid, cholesterol, and liver enzymes, reduced fetal growth, and ulcerative colitis [3,18,19,20]. This may also suggest the possibility of a PFOA role in several human pathologies such as liver diseases, cardiovascular diseases, chronic renal disease, and abnormal fetal development. Animal studies have revealed accumulation and distribution of PFOA in tissues leading to several toxicities including neurotoxicity, genotoxicity, nephrotoxicity, epigenetic toxicity, immunotoxicity, reproductive and developmental toxicity, and hepatotoxicity [21,22,23,24,25,26]. Studies have shown that PFOA can alter the activities of liver enzymes [27], cause anti-inflammatory effects by modulating the levels of proinflammatory cytokines [28], and exert cytotoxic effects on liver and thyroid cells [29,30]. PFOA has been found to cause testicular, liver, and other cancers in rats [31]. An increase in kidney and testicular cancers was also observed among people living close to chemical plants [32]. Several studies have demonstrated the alterations in metabolic pathways as a result of PFOA exposure, such as increased oxidative stress and fibrotic markers [26,33], peroxisome proliferator-activated receptor (PPARs) activation [34], initiation of P450 [35], altered expression of lipid metabolism and cell cycle genes [24], and disruption of efflux transporters—for example, Bcrp, which has an important role in the elimination of drugs and environmental chemicals from kidney and liver [36].

Through the consumption of drinking water and food contaminated with PFOA, the intestinal wall is the initial line of contact with the contaminant. PFOA was found to be stored in the intestines of various organisms such as bivalves, fishes, and crabs [37]. In vivo studies in rats revealed poor excretion of PFOA in feces which may suggest increased bioaccumulation of PFOA after repeated exposures, which may lead to significant toxicity [38]. The bioaccumulation of PFOA was found to be increased in a dose-dependent manner within the tissues of several animal models. The higher PFOA bioaccumulation was found in the intestine and liver of fishes compared to other organs such as ovary, brain, and gall bladder [37,39]. The increased bioaccumulation potential of PFASs in the intestine compared to gonads was also found in other organisms such as *Holothuria tubulosa*. Accumulation in intestinal tissue was dependent on the perfluoroalkyl chain lengths, thus PFOA and perfluorooctane sulfonate (PFOS) were found to have higher bioaccumulation potential than shorter-chain compounds [40]. It has been reported that PFOA levels in serum were positively correlated to ulcerative colitis in humans [20]. Furthermore, other commonly used PFASs, such as PFOS, were also found to alter the intestinal microbiome [41]. However, the mechanisms underlying the toxicity of PFOA in the human intestine are still unclear.

Research examining the toxic effects of PFOA in human intestinal cells is limited. It has been observed that PFOA alters the calcium homeostasis and decreased the viability of colon carcinoma (HCT116) cells over time [42,43]. Another study described the increased invasive ability of colorectal cancer cells (DLD-1 cells) via NF-κB facilitated matrix metalloproteinase-2/-9 expression after PFOA exposure [44]. This may suggest the role of PFOA in cancer metastasis. Nevertheless, although in vitro studies are common, they also raise the question as to whether in vivo models would respond in the same way to PFOA.

Despite these studies, there is still limited evidence on the toxicity of PFOA on intestinal epigenetics and tight junction (TJ) genes. From our previous studies, we observed significant alterations in mRNA expression of epigenetic genes in liver (HepG2) cells and mice kidney tissues due to acute PFOA exposure which promoted early indicators of fibroblast activation in mice kidney tissues [23,24,25,26]. The changes in the DNA methylation patterns were also found to be linked with alteration of disease-related genes in inflammatory bowel disease (IBD) and inhibition of various tumor suppressor genes in cancers [45]. Various studies published recently indicate the number of mechanisms by which epigenetic changes regulate intestinal development [46] and cause various intestinal disorders such as intestinal adenomas [47], colorectal cancer [40], intestinal inflammation [48], and inflammatory bowel disease including Crohn’s disease [49,50]. The role of epigenetics in the regulation of tight junction genes is also evident from the literature. It has been reported that metastatic and tumorigenic properties of various cancer cells were promoted by epigenetic inhibition of Occludin [51]. Similarly, DNA hypomethylation was found to cause overexpression of membranous Claudin-4 which inhibited the invasion and migration of tumor cells in gastric carcinoma [52]. These studies suggest that PFOA exposure can possibly disrupt the intestinal barrier through epigenetic alterations.

Intestinal TJs in epithelia are essential for the maintenance of the physical barrier in the intestine and regulate the flow of substances, such as solutes, ions, and water across the epithelial cells in the intestine [53]. They are also involved in various other physiological functions, from appropriate hydration to nutrient uptake, to protection against infectious agents. Several cancers and key signaling pathways involved in cell metastasis, invasion, proliferation, and transformation were found to be associated with TJ dysregulation [54]. Studies have also revealed the association of TJ dysfunction with various inflammatory and metabolic disorders, such as celiac disease, IBD, obesity, and type 1 diabetes [55,56,57,58,59]. This was further supported by the positive correlation of PFOA serum levels with the incidence of ulcerative colitis in the human population [20]. This indicates the possibility of the dissemination of PFOA and associated metabolites through a compromised intestinal barrier into different locations of the body. However, there is no study to date examining the toxic effect of PFOA on intestinal epigenetics and tight junctions.

In the current study, we examined the bioaccumulation of PFOA in the small intestine and colon tissues and the relative expression of (1) epigenetic effector genes, which are DNA methyltransferase (*Dnmt*s) and ten–eleven translocation (*Tet*s), and (2) intestinal TJ genes (i.e., occludin (*Ocln*), claudins (*Cldn*s) and zonula occludens (*ZO*s)), which have a key role in the maintenance of physical barriers in the intestine. Previous studies have mostly been conducted on colon cell lines which may not appropriately represent in vivo conditions and undermine the major anatomical differences in the intestine. Further, dose-dependent effects of PFOA have been noted in colon cancer cell lines [43]. Therefore, the focus of our work is to assess the effect of PFOA on intestinal epigenetics and tight junction genes in the small intestine and colon. We expect our findings to further assess the effect of PFOA in methylation and modulation of the intestinal barrier due to its toxicity.

## 2. Materials and Methods

### 2.1. Test Chemical and Dosing Concentrations

PFOA (C_8_HF_15_O_2_, CAS# 335-67-1, 96% purity) and Tween-20 were purchased from Sigma-Aldrich (St. Louis, MO, USA). Four different dosing concentrations 1, 5, 10, and 20 mg/kg/day of PFOA along with vehicle control (no PFOA) were prepared in 0.5% Tween-20 in deionized water. The concentrations were selected based on the reported amount of PFAS levels in the serum of people both from the environmental/community and occupational exposure. The highest concentration of PFOA reported in the blood serum of 506 fluorochemical production employees was 92.03 μg/mL with an average of 2.21 μg/mL [60]. A community exposure study showed PFOA serum concentration in the range of 7–4525 ng/mL with a primary source of PFOA in drinking water [61]. In mice studies, blood serum has been reported in a concentration range of 100–200 μg/mL dosed with 20 mg/kg/day of PFOA for different duration [62,63]. We decided our dosing concentrations (from low–high) and duration (10 days) taking into consideration the community and fluorochemical production workers’ exposure limits to PFOA.

### 2.2. Animals Housing, Dosing, and Tissues Collection

CD1 mice, an outbred strain, commonly used in toxicology were dosed to assess the adverse effect of PFOA. Animal experiments were conducted with an approved protocol (Toxicology of Endocrine Disrupting Chemicals, Protocol# 19037; Approved 4/11/2019) by the University of Illinois at Urbana-Champaign, Institutional Animal Care and Use Committee (IACUC) per National Institute of Health (NIH) guidelines. Female mice were obtained from Charles River, USA, and randomly divided into 5 groups (*n* = 3 mice per group). They were housed in polysulfone, ventilated cages at 25 °C on a 12:12 h light: dark cycle and given free access to the Teklad Rodent Diet 8604 and purified water. Mice were dosed by oral gavage after acclimatization to the new environment and reaching the age of 30 days. Different doses (1, 5, 10, and 20 mg/kg/day) of PFOA along with vehicle control were administered daily for the next 10 days.

After the completion of dosing, mice were euthanized using CO2 asphyxiation. Intestinal tissues were collected immediately after euthanasia, and intestinal contents were flushed with sterile phosphate-buffered solution (PBS). The washed tissues were stored in liquid nitrogen at the site of surgery and later transferred to a −80 °C freezer.

### 2.3. Isolation of RNA and cDNA Synthesis

Small intestine and colon tissue were processed to extract total RNA by the Trizol method (Ambion, Thermofisher, Waltham, MA, USA) and dissolved in diethyl pyrocarbonate (DEPC) treated water. The concentration and purity of RNA were analyzed by NanoDrop 1000 spectrophotometer (Thermo Fisher Scientific Inc., Waltham, MA, USA). Then, 1 μg of extracted RNA from each sample was used to transcribe cDNA using the high capacity cDNA synthesis kit (Applied Biosystems, Thermofisher, Waltham, MA, USA). The synthesized cDNA was diluted (1:20) in molecular grade water (Corning Mediatech, Inc., Manassas, VA, USA).

### 2.4. Assessing Gene Expression Variation Using Real-Time PCR

Variation in the expression pattern of specific tight junction and epigenetic genes was studied with quantitative real-time PCR (StepOnePlus Real-Time PCR Systems; v 2.0 Applied Biosystems, Waltham, MA, USA). Primers for these genes were designed and ordered using PrimerQuest Tool (Integrative DNA Technologies, Inc., Commercial Park Coralville, IA, USA). Then, 5 μL of diluted cDNA and 20 μL of Powerup SYBR Green PCR master mix (primers added) (Thermo Fisher Scientific Inc., Waltham, MA, USA) were placed in each well of the plate and qPCR was run according to their optimized annealing temperature. Glyceraldehyde 3- phosphate dehydrogenase (GAPDH) a housekeeping gene was used as an exogenous reference to normalize the transcription and the subsequent data were analyzed by the ΔΔ*C*_t_ method.

### 2.5. Quantification of PFOA Accumulated in the Intestine

PFOA extraction from the tissue was performed following the method of Mamsen et al., 2017, with some modifications [64]. Tissues were homogenized at a ratio of 10 parts 70% HPLC grade acetonitrile (in DI water) to one-part tissue using Omni THQ—Digital Tissue Homogenizer for 2 min. Samples were shaken for half an hour at room temperature, then centrifuged at 10,000× *g* and the clear supernatant containing the extracted PFOA was separated. The supernatant was diluted in 70% acetonitrile in 1:1 and then injected into Ultra Performance Liquid Chromatography–Mass Spectrometry (UPLC–MS) using SYNAPT G2-Si system (Waters Corp., Milford, MA, USA). The mobile phase used was solvent A: 5 mM Ammonium acetate (NH_4_OAc·H_2_O) and solvent B: Acetonitrile. The mobile phase was kept at 100% for 2 min, and 98% B for the next 3 min. The column was then conditioned at 100% solvent A for the last 2 min. Calibration curve was built using known concentrations of PFOA (0.78125, 0.390625, 0.195313, 0.097656, 0.048828, 0.024414, 0.012207, and 0.006104 μM) in 70% acetonitrile. Electrospray ionization (ESI) in the mass spectrometry was set in negative ion mode for PFOA detection.

### 2.6. Data Processing and Statistical Analysis

Data analysis was performed with the IBM SPSS Statistics for Windows (Version 25.0, IBM Corp., Armonk, NY, USA). Due to the relative smaller sample size, we assumed that the data were not normally distributed and therefore a non-parametric analysis, Mann–Whitney U two independent test was used. Statistical values were considered significant at *p*-value ≤ 0.05 (denoted by “*”) and less significant at *p*-value > 0.05 but < 0.1 (denoted by “^”) reported from two-tailed tests.

## 3. Results

### 3.1. Accumulation of PFOA in the Intestinal Tissues

**Figure 1:**
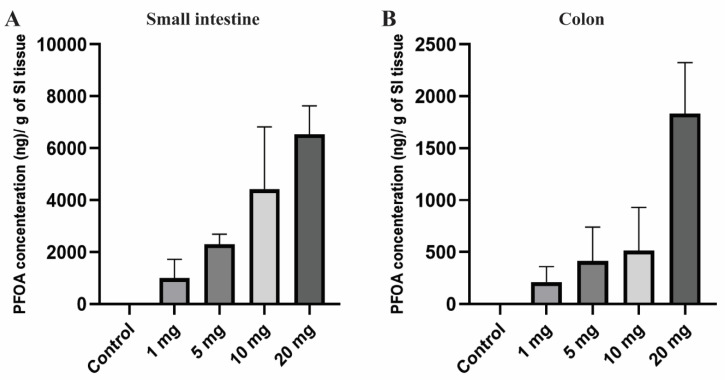
Bioaccumulation of PFOA in the intestinal tissues of CD1 mice. Mice were treated with control (0.5% Tween-20 in water) or 1–20 mg/kg/day PFOA for 10 days. (**A**) Quantification of PFOA in ng/gram in small intestine tissue; (**B**) quantification of PFOA in ng/gram of colon tissue using UPLC chromatography.

PFOA, the persistent perfluoroalkyl substance, has a high potential to accumulate in human and animal bodies and shows a large variation in concentration across the different tissues [65]. Extended half-life and high bio-accumulative characteristics increased its toxicity in living organisms [17]. After 10 days of PFOA exposure, small intestine and colon tissues of the female CD1 mice were noted to have a significant amount of PFOA accumulation compared to the control group in which no PFOA was detected. Accumulation of PFOA gradually increased in the tissue with an increase in dosing concentrations (Figure 1). The mean concentration of PFOA detected was 1002.62, 2298.06, 4427.71, and 6533.69 ng/g in the small intestine and 211.12, 412.73, 513.45, and 1834.27 ng in colon tissue for the treatment groups 1, 5, 10, and 20 mg/kg/day, respectively.

Overall, there was a very high dose-dependent bioaccumulation rate in intestinal tissues with much higher bioaccumulation in the small intestine compared to the colon, which may suggest dose-dependent effects of PFOA on gut epigenetics and permeability explored in the current work.

### 3.2. PFOA Induced Alteration of the Epigenetic Regulators

**Figure 2:**
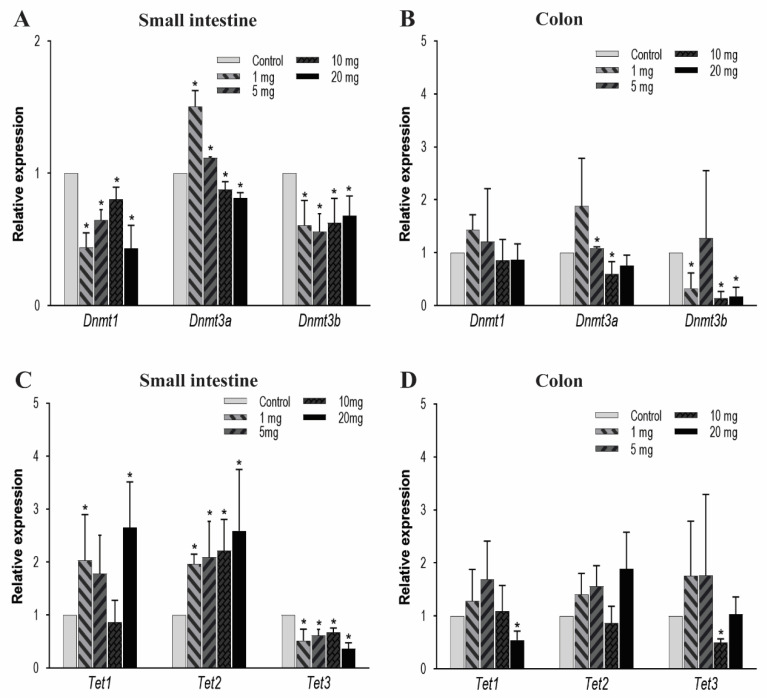
Effect of PFOA on the expression levels of DNA methylation (*Dnmts*) and DNA demethylating genes (*Tets*) in intestinal tissues of CD1 mice. Mice were treated with control (0.5% Tween-20 in water) or 1–20 mg/kg/day PFOA for 10 days. (**A**) Change in the mRNA expression level of *Dnmt1*, *Dnmt3a* and *Dnmt3b* genes in the small intestine; (**B**) change in the mRNA expression level of *Dnmt1*, *Dnmt3a* and *Dnmt3b* genes in the colon; (**C**) change in the mRNA expression level of *Tet1*, *Tet2* and *Tet3* genes in the small intestine; (**D**) change in the mRNA expression level of *Tet1*, *Tet2* and *Tet3* genes in the colon. Statistical values were considered significant at *p*-value ≤ 0.05 (denoted by “*”) and less significant at *p*-value > 0.05.

The DNA methylation is predominantly regulated by DNMT and TET enzymes. DNMTs have various isoforms and in the current study, we primarily focused on DNMT3a, DNMT3b, and DNMT1. These isoforms were principally selected because in the early development, the de novo DNMTs (i.e., DNMT3a and DNMT3b) play an important role in forming the 5 mC patterns in the genome and these genomic patterns were then maintained by DNMT1. In the small intestine, there is a significant decrease in the expression pattern of the genes *Dnmt1* and *Dnmt3b* in all of the treatment groups (Figure 2A). *Dnmt3a* was expressed more in low dosing groups: 1 and 5 mg/kg/day and under-expressed at higher concentrations of 10 and 20 mg/kg/day. In colon tissues, we observed that after PFOA exposure the *Dnmt3b* expression level in higher concentrations was significantly reduced and the *Dnmt3a* expression level for the 10 mg/kg PFOA exposure was also found to be significantly reduced; hence, affecting the DNA methylation levels in the colon. Unlike *Dnmt3b* and *3a*, *Dnmt1* had no significant change in its expression level when exposed to increasing concentrations of PFOA (Figure 2B).

Next, we evaluated the mRNA expression levels of TETs enzymes. TETs are a family of three protein enzymes, which include TET1, TET2, and TET3. These three enzymes catalyze a set of reactions which include oxidation of 5 mC to 5 hmC, 5-formylcytosine, and then finally to 5-carboxylcytosine [66,67], therefore resulting in DNA demethylation. In the small intestine, *Tet1* expression increased significantly at low and high concentrations, while *Tet2* shows a significant increase at all dose levels. In contrast, *Tet3* expression reduced significantly in all treatment groups (Figure 2C). In colon tissues, the mRNA expression for *Tet3* significantly decreased after exposure to 10 mg/kg of PFOA, and *Tet1* expression decreased after exposure to 20 mg/kg only. No significant change in mRNA expression of *Tet2* after exposure to PFOA (Figure 2D) was noted. Therefore, our data suggest that at higher concentrations a concentration-dependent effect on the mRNA expression of key enzymes that regulate DNA methylation is possible, suggesting strong evidence of PFOA-induced epigenetic effects in colon tissue.

### 3.3. PFOA Induced Alteration of Tight Junction Genes

#### 3.3.1. mRNA Expression Analysis of Cldns Genes

**Figure 3:**
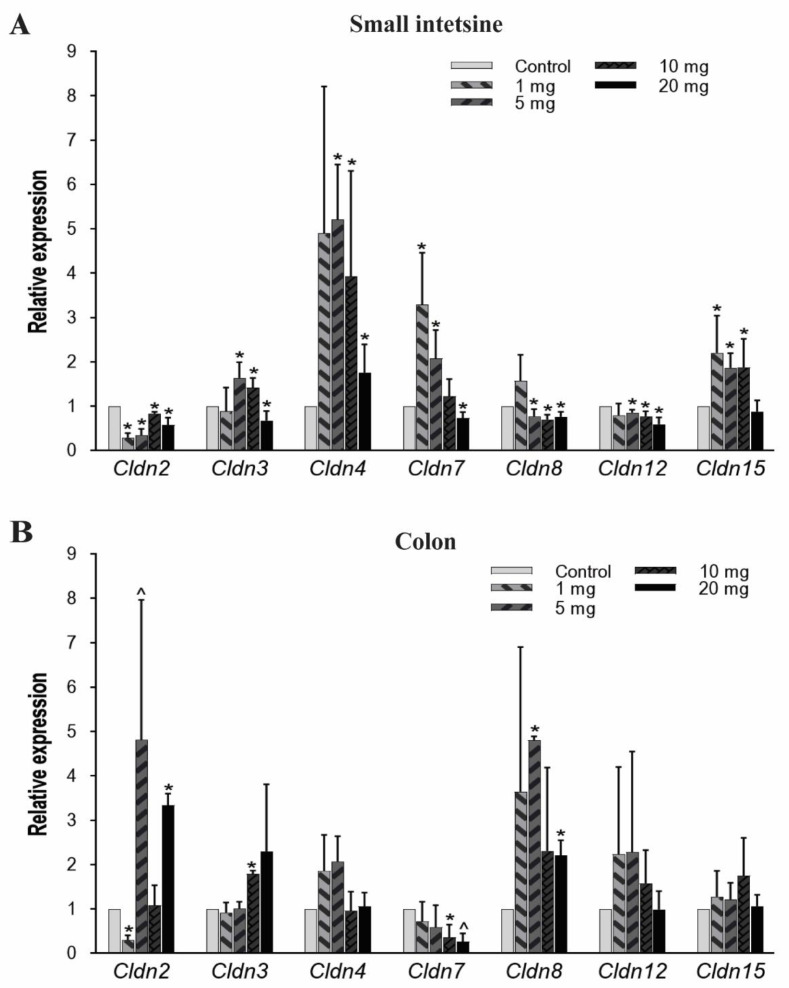
Effect of PFOA on the expression levels of tight junction gene *Cldn* in intestinal tissues of CD1 mice. Mice were treated with control (0.5% Tween-20 in water) or 1–20 mg/kg/day PFOA for 10 days. (**A**) Change in the mRNA expression level of *Cldn2, Cldn3, Cldn4, Cldn7, Cldn8, Cldn12,* and *Cldn15* genes in the small intestine; (**B**) change in the mRNA expression level of *Cldn2, Cldn3, Cldn4, Cldn7, Cldn8, Cldn12*, and *Cldn15* genes in the colon. Statistical values were considered significant at *p*-value ≤ 0.05 (denoted by “*”) and less significant at *p*-value > 0.05 but < 0.1 (denoted by “^”) reported from two-tailed tests.

The structural integrity, barrier and transport functions of the gut epithelial lining are primarily regulated by tight junction proteins [54]. In the current study, we examined seven members of *Cldn* (transmembrane protein family), which were *Cldn2, Cldn3, Cldn4, Cldn7, Cldn8, Cldn12,* and *Cldn15*. In the small intestine, *Cldn2* mRNA expression significantly decreased at all PFOA dosings, while *Cldn8* and *Cldn12* showed low expression at 5, 10, and 20 mg/kg/day doses. *Cldn4* expression significantly increased at exposure levels of 5 mg/kg and gradually decreased with an increase in PFOA concentration but still higher than the untreated/control mice. Likewise, the *Cldn3* gene has a higher expression at 5 and 10 mg/kg, but the mRNA level decreased at 20 mg/kg compared to control. *Cldn15* expression pattern was significantly elevated at 1, 5 and 10 mg/kg/day treatment groups whereas *Cldn7* expressed more at lower doses and then significantly decreased in expression at 20 mg/kg/day (Figure 3A).

In colon tissues, *Cldn 2* expression was found to be significantly increased when exposed to the highest concentration (20 mg/kg) of PFOA. Similarly, *Cldn 3* and *Cldn 8* mRNA expression levels also increased at higher concentrations. However, *Cldn 7* expression level was reduced at higher PFOA dose levels. Unlike *Cldn 2, Cldn 3, Cldn 7,* and *Cldn 8,* other *Cldns* (*Cldn 4*, *Cldn 12,* and *Cldn 15*) were not affected by the exposure (Figure 3B).

#### 3.3.2. mRNA Expression Analysis of *Tjps* and *Ocln* Genes

**Figure 4:**
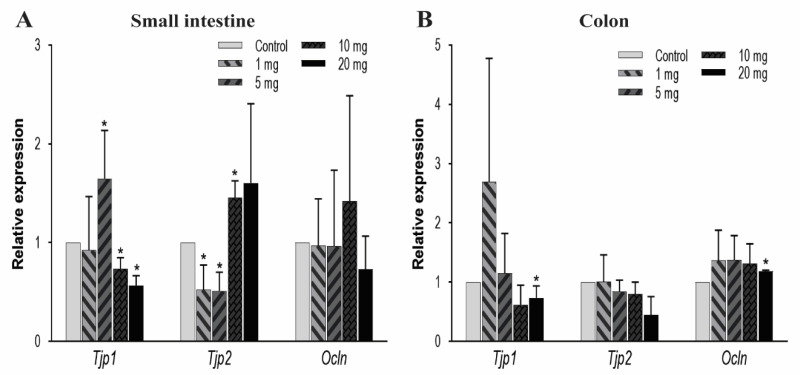
Effect of PFOA on the expression levels of tight junction genes *Tjps* and *Ocln* in intestinal tissues of CD1 mice. Mice were treated with control (0.5% Tween-20 in water) or 1–20 mg/kg/day PFOA for 10 days. (**A**) Change in the mRNA expression of *Tjp1, Tjp2,* and *Ocln* genes in the small intestine; (**B**) change in mRNA expression of *Tjp1, Tjp2*, and *Ocln* genes in the colon. Statistical values were considered significant at *p*-value ≤ 0.05 (denoted by “*”) and less significant at *p*-value > 0.05.

To further explore the effect of other TJ components on intestinal permeability, we observed the mRNA expression levels of zonula occludens, i.e., TJ proteins (*Tjp-1* and *Tjp-2*) and occludin (*Ocln*) genes. In the small intestine, *Tjp1* was significantly upregulated at 5 mg/kg/day of PFOA dosing but gradually decreased in expression at higher levels of exposure. Conversely, *Tjp2* expression was very low at 1 and 5 mg/kg/day and high at an exposure level of 10 mg/kg/day, whereas *Ocln* expression remained unaffected (Figure 4A). In the colon, the mRNA expression levels for *Tjp1* and *Ocln* were found to decrease at 20 mg/kg concentration. Whereas, *Tjp2* expression remained unaffected due to PFOA exposure (Figure 4B). These expression profiles showed a significant effect of PFOA on these protective barriers to indicate a possible dysregulation in the linked pathways.

## 4. Discussion

PFOA is a commonly used synthetic chemical that has a high persistence and bioaccumulation in environmental media and the human body [13]. Recently, another commonly used PFAS compound, perfluorooctane sulfonate (PFOS) was found to disrupt the intestinal microbiome in mice, causing intestinal tissue damage [41]. In the current study, we expect that PFOA exposure could compromise the expression of epigenetic genes and intestinal barrier function in mice.

### 4.1. Orally Administered PFOA Accumulates in the Intestine

The intestinal wall is an initial contact barrier to environmental chemicals after being ingested through food or drinking water. PFOA has a reduced elimination rate in feces which may lead to its bioaccumulation in various organs including the intestine [38]. In our experiments, a significant dose-dependent increase in PFOA concentration was observed in the small intestine and colon tissues (Figure 1). Higher levels of accumulation were noted in the small intestine compared to colon tissues. In vivo exposure studies conducted in the past have also demonstrated a significant amount of PFOA, as well as the accumulation of other PFAS compounds in intestinal tissues compared to other organs [37,39,40]. The current study is in line with the studies conducted in the past.

### 4.2. PFOA Alters Expression of DNA Methylation Genes in the Intestine

To explore the epigenetic mechanisms that can cause gene expression changes in the small intestine and colon tissues exposed to PFOA, we specifically studied the mRNA expression changes of genes involved in DNA methylation mechanisms. Various studies published recently indicate a number of mechanisms by which epigenetic changes regulate intestinal development [46] and cause various intestinal disorders [40,48], thus providing the opportunities to identify new epigenetic biomarkers as treatment targets.

There are a limited number of studies available that explore the epigenetic alterations in the intestine due to PFOA exposure. The studies available are mostly performed on blood samples collected from the PFOA-exposed population [68,69]. One of these studies has revealed the hypomethylation of glutathione-S-transferase Pi in liver cells [70]. The previous studies from our group have also reported significant alterations in DNA methylation in vitro in HepG2 cells [24] and in vivo in mice kidney and liver tissues [23,26]. However, the effect of PFOA on the epigenetic alterations of intestinal tissues has not been studied to date.

For examination of the mRNA expression levels of *Dnmt*s and *Tet*s, key regulators of DNA methylation and demethylation, respectively, we observed a significant decrease in the mRNA expression levels of *Dnmt1* and *Dnmt3b* in small intestine tissue. *Dnmt3a* level was found to be significantly reduced when exposed to higher concentrations of PFOA (i.e., 10 and 20 mg/kg), thus affecting the de novo methylation of DNA and maintenance of these genomic patterns (Figure 2A). Similarly, in colon tissues, *Dnmt3b* expression levels were found to be reduced when exposed to 1, 10, and 20 mg/kg of PFOA for 10 days, whereas the *Dnmt3a* level was found to be reduced only when exposed to 10 mg/kg of PFOA. No significant changes were observed for the *Dnmt1* levels (Figure 2B). This may suggest the effect of PFOA on de novo methylation in the colon, but the maintenance of these DNA methylation patterns in the colon may remain unaffected due to insignificant changes in *Dnmt1* levels. However, these could be explored in the future with further experiments. More severe effects of the expression levels of *Dnmts* in the small intestine could be due to a higher accumulation of PFOA in the small intestine compared to the colon especially at higher dose levels. Nevertheless, the reduction in the expression of *Dnmts* can possibly have a beneficial effect in preventing diseases such as colorectal carcinoma by reactivation of the tumor suppressor genes in the intestinal tissue [71].

The demethylation of CpG is a result of hydroxymethylation, which is mediated by ten–eleven translocation enzymes (TET1, TET2, and TET3). Past studies have demonstrated the reduced mRNA expression of *Tet1* in intestinal tumors and gastric cancers [72,73]. Further, *Tet3* expression was found to be reduced in the disease model of experimental fibrosis, whereas *Tet2* expression was not found to be significantly reduced in disease conditions [74]. Our study also reveals reduced expression of *Tet3* in the small intestine in all the treatment groups and colon tissues from the 10 mg/kg treatment group. The *Tet1* expression in the colon was also found to be reduced in the 20 mg/kg treatment group (Figure 2D). This reduction in expression levels of *Tet3* and *Tet1* could be due to the result of a decrease in the levels of *Dnmts* to balance the net amount of DNA methylation within the tissue. However, we observed an increase in *Tet1* and *Tet2* expression levels in small intestinal tissues after PFOA exposure (Figure 2C) that may potentially lead to change in expression levels of other functional genes in the small intestine and colon. Alterations in the expression of epigenetic regulators seem to be more profound in the small intestine, possibly due to the higher accumulation of PFOA compared to the colon. These evaluations suggest that direct environmental and occupational exposure to PFOA can possibly lead to epigenetic alterations in the intestinal tissues. These methylation anomalies can be of potential significance in the development of human pathologies as is evident from previous studies in animal models, which showed their association with inflammatory bowel disease (IBD) [45], compromised intestinal development [46], intestinal adenomas [47], colorectal cancer [40], intestinal inflammation [48], and inflammatory bowel disease including Crohn’s disease [49,50]. The epigenetic changes can also lead to overexpression and inhibition of various TJ genes which can promote tumorigenic and metastatic properties of gastric cancer cells [51,52]. Future work can focus on evaluation of methylation levels of TJ genes and other key signaling pathways involved in intestinal tumors in PFOA-exposed mice to explore the direct effect of methylation changes in the development of intestinal pathologies.

### 4.3. PFOA Alters Expression of TJ Genes in the Intestine

As the gastrointestinal tract (GIT) is the primary route of exposure to the PFOA present in the drinking water and food chain, it can affect the structural integrity and function of the gut epithelial lining. The integrity and barrier function of the gut lining are maintained by tight junction proteins that seal the adjacent cells to regulate transport and avoid leakage [75]. Tight junction proteins either localize as transmembrane proteins such as *Cldn* and *Ocln* or cytoplasmic proteins, *Tjp* [76,77]. Dysfunctionality in these proteins has been implicated in several disorders, as in impaired blood–brain barrier and blood–retinal barrier, inflammatory bowel disease, breast cancer, celiac disease and even in heredity diseases such as deafness [78,79,80].

Several studies have shown an increase in permeability of the gut due to environmental inducers such as synthetic food additives (emulsifiers and surfactants), nanoparticles, and alcohol which disrupt tight junction proteins that lead to leaky gut [81,82]. Further, many viral and bacterial infections are known to destroy these junction proteins and cause leakage in epithelial linings [83,84]. The adverse effects of environmental toxicants on the tight junction proteins are an unexplored area. There is a need to evaluate the GIT integrity and function in PFAS-exposed tissue to mimic the exposure route of these toxicants through water and food. Therefore, we examined the variation in the mRNA expression pattern of key tight junction genes (*Cldn, Ocln,* and *Tjp*) in the small intestine and colon tissue in orally administered mice with PFOA.

The results from the present study show that PFOA had an effect on these tight junction genes and there is a substantial change in mRNA expression between the control and treatment group mice. Further, a variation in the expression pattern between the small intestine and colon suggests that these tissues are affected differently by PFOA treatment. We examined seven members of the *Cldn* protein family, which either functions as a paracellular barrier or channel depending upon the extracellular regions of these proteins [85,86]. In the small intestine, expression of *Cldns* was significantly affected, in proportion to the PFOA concentration and type of *Cldn* member. The change in the expression pattern can be linked to the elevated level of PFOA accumulation in the small intestine. Further, *Dnmts* are previously reported to be linked with the change in the expression profile of *Cldn* in different carcinomas [52,87]. This is an indication that the heavily altered expression pattern of *Dnmts* in the current study would have affected *Cldn* transcription in the small intestine. In the colon, *Cldn 2, Cldn 3,* and *Cldn 8* expression was greater at higher levels of exposure to PFOA. Since PFOA was detected in the colon in high amounts, especially in the higher concentration treatment groups, its effect was proportional to the expression pattern of some of the *Cldns*.

Occludin *(Ocln),* a transmembrane tight junction that is indirectly involved in the barrier function through regulation of other tight junction proteins [57], remained unchanged in the small intestine whereas in the colon it was downregulated at the highest PFOA dose level. The knockout of the *Ocln* gene in mice model has shown chronic inflammation and poor tight junction integrity [88]. *Tjps* genes, which encode the cytoplasmic proteins to bridge transmembrane and cytoskeletal proteins [57], showed dysregulation in mRNA expression across the intestine. Mutation in *Tjp2* inhibits its linkage to *Cldn* and leads to biliary disease [89]; while knockdown of the *Tjp1* gene induces the condition of leaky gut [90].

The change in the expression profile of tight junction genes was more conspicuous in the small intestine compared to the colon. Our results show that PFOA accumulation was greater in the small intestine compared to the colon which is a distal part of the GIT. Further, it is evident from our previous study that PFOA accumulation was almost six-fold more in the liver compared to the small intestine [23]. Our results suggest that orally administered PFOA accumulates in the small intestine and is absorbed in higher levels from here to the liver and in trace amounts as it passes to the distal colon. Hence, more significant changes in gene expression were observed in the small intestine than in the colon.

## 5. Conclusions

In the current study, the expression of epigenetic and tight junction genes was found to be altered in intestinal tissues with more profound effects in the small intestine due to increased accumulation of PFOA. The alterations in the expression levels of tight junction genes evaluated may be due to the epigenetic toxicity at high concentrations of PFOA found in the intestinal tissues. Nevertheless, further research is needed to examine the methylation profiles of the tight junction genes to confirm the direct epigenetic effect of PFOA on the regulation of genes expressed in intestinal tissues. It would also be prudent to assess how sub-acute and chronic PFOA exposure could affect epigenetic factors and the pathways triggered. Transgenerational studies could provide valuable insights on how these effects are propagated.
